# Sociodemographic factors and delay in the diagnosis of cervical cancer in Morocco

**Published:** 2012-05-25

**Authors:** Mohamed Berraho, Majdouline Obtel, Karima Bendahhou, Ahmed Zidouh, Hassan Errihani, Abdellatif Benider, Chakib Nejjari

**Affiliations:** 1 Laboratoire d'Epidémiologie, Recherche Clinique et Santé Communautaire; Fès, Maroc; 2 Equipe Epidémiologie de la Prévention des Cancers, INSERM 897 ISPED; Bordeaux, France; 3 Association Lalla Salama de Lutte contre le cancer (ALSC), Maroc; 4 Institut National d'Oncologie (INO) – Rabat, Maroc; 5 Centre d'oncologie, Centre Hospitalier Universitaire Casablanca, Maroc

**Keywords:** Cervical cancer, delay, late diagnosis, Morocco

## Abstract

**Background:**

In Morocco, cervical cancer is the second most common cancer in women. The cases of cervical cancer are diagnosed at a late stage: 43.7% presented at stage II of diagnosis (FIGO) and 38.1% in advanced stage (stage III and IV). The main objective of this study is to investigate factors associated to late the diagnosis of cervical cancer in Morocco as measured by the stage at diagnosis and delays between first symptoms and diagnosis of cancer.

**Methods:**

Cross-sectional studies, conducted from June-2008 to June-2010 at two main oncological centers. Two-hundred cases were recruited. Stages I & II were identified as “early-stage”. The dates of first-symptoms, first-consultation and first-diagnosis were used to define “Patient”, “Medical” and “Total” delays.

**Results:**

Elevated risks for late stage was observed for women unmarried (OR=5.0; 95%CI: 1.43-16.66); living > 100 km from center of diagnosis (OR=4.51; 95%CI: 1.35-15.11); without a familial history of cancer (OR=14.28; 95%CI: 2.22-100) and whose was the first symptom not bleeding (OR=25; 95%CI: 1.62-300). Frequency of housewives was significantly higher for women with a “patient-delay” ≥1 month. Frequency of patients who had symptoms of “bleeding” was significantly higher for women with a “patient-delay” <1 month. Frequency of patients from urban area was significantly higher for women with a “Medical-delay” < 1 month. Elevated risks for a long “Total-delay” was observed for women aged < 50 years (OR=2.44; 95%CI: 1.24-4.76); illiterate (OR = 3.85; 95%CI: 1.45-10.00) and from rural-area (OR=2.56; 95%CI: 1.25-5.26).

**Conclusion:**

Our results may represent an important tool in guiding the actions for an early diagnosis of cervical cancer.

## Background

Cervical cancer is the second most common cancer among women worldwide [1]. It accounted for an estimated 529 409 incident cases, for a world age standardized incidence rate 15.2 incident cases per 100000 women per year, and 274 883 deaths in the world in 2008 [2], constituting approximately 8% of the global burden of cancer among women [2]. Cervical cancer continues to be a disease related to socio-economic and demographic (SEDS) disparities in both developing as well as developed countries. In the U.S., despite the overall downward trend in cervical cancer there still exists a disparity in mortality rates for cervical cancer related deaths among certain ages as well as racial, geographic and socio-economic groups. It has been found that race, ethnicity, age greater than 65 years, lower education, lack of health coverage, and rural location are associated with inadequate preventive cervical cancer screening [3, 4]. Analyses of the USA cancer data have shown that mortality due to cervical cancer increases with poverty and decreasing education. In addition, a negative correlation exists between socio-economic groups and stage at diagnosis [5]. Studies have shown that late stage at diagnosis is correlated with lower survival rates in cervical cancer patients [6]. It has also been reported that longer durations of symptoms in cervical cancer patients as well as of treatment prolongation negatively affect survival [7, 8]. These two factors can therefore be useful predictors for the severity of illness and likelihood of survival.

In Morocco, cervical cancer is the second most common cancer of women after breast cancer [9]. According to GLOBOCAN 2008 [2], the world age-standardized incidence of cervical cancer among women in Morocco was 14.1 new cases/100 000 inhabitants / year (1979 new cases/year). The mortality rate from this cancer was 8.4 per 100 000 (1152). The stage of diagnosis is the most important independent factor of prognosis [10, 11]. In Morocco, a recent study [12] has been conducted with the aim to provide descriptive epidemiological and pathological characteristics of cervical cancer among patients attending the most important oncology centers of Morocco; this study showed a late diagnosis of cervical cancer in Morocco. Indeed, 43.7% were presented with stage II at diagnosis II (FIGO) and 38.1% were presented in advanced stages (stages III and IV). The main objective of this study is to investigate the factors associated with delayed diagnosis of cervical cancer in Morocco as measured by the stage at diagnosis and delays between first symptoms and diagnosis of cancer.

## Methods

### Population and study sites

This is a cross-sectional study with consecutive recruitment from June 2008 to June 2010 of patients with cervical cancer at the National Institute of Oncology in Rabat and the Oncology Hospital of the University Hospital Ibn Rushd of Casablanca.

### Data collection

The information was collected by previously trained interviewers using a standardized collection sheet. This information has been filled by administering questions face to face with patients and supplemented from their medical records. The record collection includes patient demographic data (age, sex, origin, educational level and area of residence, etc ....), data on personal and family history of cancer, data on classification of disease (FIGO) and the dates of the various points in the history of the disease: date of first symptoms, date of first consultation and the date of first diagnosis. Stages I and II were identified as “early stage” and III and IV as “late stage”. The date of first symptoms, date of first consultation and date of first diagnosis were collected. Three indicators of delay were considered: Patient delay” time, Medical delay” time and Total delay time. Patient delay time was defined as the interval between the date of first symptoms and the date of first consultation. The “Medical delay” time was defined as the interval between the date of first consultation and the date of first diagnosis. The “Total delay” time was defined as the interval between the date of first symptoms and the date of first diagnosis.

### Statistical analysis

The analysis involved a description of the study population according to different sociodemographic and socioeconomic characteristics. Chi2 tests, Student test and Fisher test were used to identify factors associated with different delays. Logistic regression was used to identify factors associated with late stage and total delay. A P-value of <0.05 was used to indicate statistical significance. All analyses were performed with Epi-info (Version 3.4).

## Results

Table 1 describes different demographic, socioeconomic and medical characteristics of the patients and their relation to the stage at diagnosis. The total number of patients analyzed was 200. Patient age ranged from 28 to 83 years with a mean age of 51.98 years (SD=12.37 years). Nearly fifty seven percent of patients were aged 50 years and over. Nearly sixty-one percent of patients resided in urban area. Nearly fifty seven percent of patients were married. Patients in the lowest income category represent 88.5% and medium income 11.5%. Nearly eighty percent of patients were illiterate and 20.5% had primary or middle school level. Almost ninety-five percent of patients identified their occupation as ‘housewife’. Only 5.3% of the patients had a health insurance and 59.7% lived at a distance =100 Km of the Oncology center. Only 16.8% of the patients had antecedents of cancer and 89.6% had consulted for gynecological bleeding.

**Table 1:** Distribution and Association of Socioeconomic and Demographic Factors by Stage at Diagnosis: Multivariate Analysis

	Total		Stage of cancer				Logistic regression
	N	%	Early	Late	OR	95% CI	*P*
**Age (years)**							
<50	74	43.3	49.4	38.3	1	–	–
≥50	97	56.7	50.6	61.7	0.73	0.21–2.56	0.62
**Occupation**							
Housewife	167	94.9	93.8	95.8			
Other	9	5.1	6.3	4.2			
**Marital status**							
Married	99	56.6	53.8	58.9	1	–	
Other§	76	43.3	46.3	41.1	5.00	1.43–16.66	0.01
**Educational level**							
Educated	35	20.5	26.0	16.0			
Illiterate	136	79.5	74.0	84.0			
**Residence area**							
Urban	121	60.8	60.0	56.3			
Rural	78	39.2	40.0	43.8			
**Socioeconomic level**							
Medium	20	11.5	15.2	8.4	1	–	
Low	154	88.5	84.8	91.6	5.26	0.78–50.00	0.09
**Health insurance**							
Yes	9	5.3	5.2	5.3			
No	162	94.7	94.8	94.7			
Distance*							
<100 Km	60	40.3	44.3	37.5	1	–	
≥100 Km	89	59.7	55.7	62.5	4.51	1.35–15.11	0.01
History of cancer**							
Yes	29	16.8	25.3	9.6	1	-	
No	144	83.2	74.7	90.4	14.28	2.22–100	0.005
Bleeding***							
Yes	172	89.6	93.9	87.3	1	–	
No	20	10.4	6.1	12.7	25.00	1.62–333	0.02

§ Single, widowed and divorced

* Distance between residence area and place of diagnosis

** History of familial antecedent of cancer

*** Bleeding as first gynecological symptom

### Stage of diagnosis

In our population, 45.5% were diagnosed at an early stage of cervical cancer and 54.5% were diagnosed at a late stage. Nearly sixty-two percent of women with late stage of diagnosis were aged 50 years or older vs. 50.6% of women with early stage of diagnosis. Nearly ninety-two percent of women with late stage of diagnosis had low socioeconomic level vs. 84.8% of women with early stage of diagnosis. Eighty-four percent of women with late stage of diagnosis were illiterate vs. 74.0% of women with early stage of diagnosis. Nearly eighty-seven percent of women with late stage of diagnosis had bleeding as first gynecological symptom vs. ninety-four of women with early stage of diagnosis. In the multivariate analysis, elevated risks for late stage at cancer reporting were associated with three variables: marital status, distance from oncology center and gynecological bleeding. Women who were not married (OR=5.0; 95% CI: 1.43-16.66); women who live more than 100 km from the location of cancer diagnosis (OR=4.51; 95% CI: 1.35-15.11); women without a family history of cancer (OR=14.28; 95% CI: 2.22-100) and women who had other signs that the bleeding as the first symptom (OR=25; 95% CI: 1.62-300) were more at risk of late stage cancer (Table 1).

### Patient delay

Table 2 describes demographic and socioeconomic factors and their relation to “Patient delay” and “Medical delay”. “Patient delay” duration was divided into two groups, less than one month, and equal to longer than one month. Nearly sixty percent of women had a “Patient delay” equal or more than one month. Three variables were associated with a patient delay = 1 month: occupation, educational level and gynecological bleeding. Frequency of housewives was higher in the class =1month (96.4%) than in the class < 1 month (89.4%) (P=0.05). Frequency of patients who had symptoms of “bleeding” or “bleeding with other symptoms” was higher in the class <1 month (97.9%) than in the class =1month (86.0%) (P=0.02). For educational level we found a trend although there was no statistically significant association. Indeed, the frequency of illiterate patients was higher in the class =1month (84.3%) than in the class

**Table 2:** Distribution and association of socioeconomic, demographic Factors and symptoms by “Patient delay” and “Medical delay”

	Patient delay (months)			Medical delay (months)		
	<1	≥1	*P*	<1	≥1	*P*
**Age (years)**						
<50	47.9	43.6	0.56	40.7	53.5	0.08
≥50	52.1	56.4		59.3	46.5	
**Occupation**						
Housewife	89.3	96.4		93.0	94.6	
Other	10.7	3.6	0.05	7.0	5.4	0.34
**Marital status**						
Married	58.7	52.3	0.39	53.1	60.8	0.29
Other§	41.3	47.7		46.9	39.2	
**Educational level**						
Educated	26.0	15.7		20.0	16.4	
Illiterate	74.0	84.3	0.08	80.0	83.6	0.54
**Residence area**						
Urban	64.5	57.3	0.32	67.3	52.7	0.045
Rural	35.5	42.7		32.7	47.3	
**Socioeconomic level**						
Low	87.8	90.7	0.52	89.2	91.8	0.56
Medium	12.2	9.3		10.8	8.2	
**Health insurance**						
Yes	8.0	7.5	0.89	5.5	9.5	0.30
Now	92.0	92.5		94.5	90.5	
Distance*						
<100 Km	40.3	38.9	0.86	37.3	43.9	0.41
≥100 Km	59.7	61.1		62.7	56.1	
Antecedent of cancer**						
Yes	13.3	15.0		16.1	11.1	0.34
Now	86.7	85.0	0.75	83.9	88.9	
Bleeding***						
Yes	97.9	86.0	0.02	92.0	90.9	0.56
Now	2.1	14.0		8.0	9.1	

§ Single, widowed and divorced

* Distance between residence area and place of diagnosis

** History of familial antecedent of cancer

*** Bleeding as first gynecological symptom

### Medical delay

“Medical delay” duration was divided into two groups, less than one month, and equal to longer than one month. Nearly sixty-one percent of women had a “Medical delay” equal to longer than one month. Only one variable was associated with a medical delay =1month: residence area. The frequency of patients from urban area was higher in class <1 month (67.3%) than in the class =1month (52.7%) (P=0.045). Nevertheless we also found a trend for age although there was no statistically significant association. Indeed, the frequency of patients aged 50 years and above was higher in the class <1 month (59.3%) than in the class =1month (46.5%) (P=0.08) (Table 2).

### Total delay

“Total delay” duration was divided into two groups, less than six months, and equal or more than six months. Nearly thirty-six percents of women had a “total delay” equal or more than six month vs. sixty-four percent of patients with □total delay” less than six months. In the multivariate analysis, three variables were significantly associated with a total delay =6 months: age, educational level and residence area. An elevated risks for high “total delay” (=6 month) was observed for women who were aged under 50 years (OR=2.44; 95% CI: 1.24-4.76); illiterate women (OR=3.85; 95% CI: 1.45-10.00) and women from rural area (OR=2.56; 95% CI: 1.25-5.26) (Table 3).

**Table 3:** Distribution and association of socioeconomic, demographic Factors and symptoms by “total delay”

	Univariate analysis			Logistic regression		
	<6mois	≥6mois	*P*	OR	95% CI	*P*
**Age (years)**						
<50	38.9	57.1	0.02	1	-	
≥50	61.1	42.9		2.44	1.24-4.76	0.01
**Occupation**						
Housewife	92.2	95.3				
Other	7.8	4.7	0.32			
**Marital status**						
Married	55.7	56.3	0.93			
Other§	44.3	43.8				
**Educational level**						
Educated	23.9	11.5	0.04	1	-	
Illiterate	76.1	88.5		3.85	1.45-10.0	0.007
**Residence area**						
Urban	73.4	57.0	0.03	1	-	
Rural	26.6	43.0		2.56	1.25-5.26	0.01
**Socioeconomic level**						
Low	88.4	93.7	0.25			
Medium	11.6	6.3				
**Health insurance**						
Yes	8.7	5.0	0.28			
Now	91.3	95.0				
Distance*						
<100 Km	38.8	40.7	0.81			
≥100 Km	61.2	59.3				
Antecedent of cancer**						
Yes	13.3	12.9	0.94			
Now	86.7	87.1				
Bleeding***						
Yes	93.1	87.5	0.28			
Now	6.9	12.5				

§ Single, widowed and divorced

* Distance between residence area and place of diagnosis

** History of familial antecedent of cancer

*** Bleeding as first gynecological symptom

## Discussion

The present study analyzed the impact of sociodemographic and socioeconomic factors among cervical cancer patients in relation to the stage at diagnosis, patient delay, medical delay and total delay. It showed mainly a problem of late diagnosis of cervical cancer and allowed to identify factors associated with delays before the diagnosis of cervical cancer. Our study shows that the housewives and patients with first symptoms other than bleeding had a high risk of long “patient delay”. Also, the long “Medical delay” was higher in the rural area and the “total delay” was higher for women aged less than 50 years and for illiterate women.

In our population 54.5% were diagnosed at a late stage of cervix cancer. In fact, the stage at diagnosis is the most important independent prognostic factor for mortality [10, 11]. Indeed, the survival rate at 5 years decreases with stage: 85% for stage IB to 0-20% for stage IVA [13, 14]. The mortality rate from cervical cancer is highly dependent on stage at diagnosis. Younger women are more likely to be diagnosed with localized cancer that carries a good prognosis [15]. Similarly, the risk of pelvic recurrence increases with stage, 10% for stage IB to more than 75% for stage IVA [2, 9]. Finally, the risk of distant metastases also increases with stage, respectively 16%, 26%, 39% and 75% for stage I, II, III and IV [16].

Patients were more likely diagnosed as having a late stage of cancer if they were not married. That may be due to absence of support from the family and this absence may discourage patients from consulting early. One reason for this may be that emotional support attributed to a husband may enable and even promote women to consult early.

In our population elevated risks for late stage were observed for women who live more than 100 km from the location of a cancer diagnosis center. Indeed access to care is an important element in decision making for consultation and thus diagnosis of cancer. For this population, in addition to the costs of examination counseling and treatment there are added costs and difficulties linked to travel, which represents an economic burden for this population; this is especially true in our population characterized for a large part by a low socioeconomic level. Indeed, only 5.3% of patients had a health insurance, 88.5% had a low socioeconomic level, only 9.0% had a professional activity and 91.0% were housewives.

In our study elevated risks for late stage reporting were observed for women without a familial history of cancer. This may be explained by the fact that women with a family history of cancer are more aware and thus more motivated to consult earlier and thus be diagnosed at earlier stages.

In our study, women who had gynecological bleeding as the first symptom had a lower risk for being diagnosed at a late stage. This can be explained by the fact that gynecological bleeding is usually perceived as more urgent and more serious than gynecological infection or pelvic pain. Thus, it represents a pattern of faster consultation than other gynecological signs. This is truer considering that the mean age in our population is almost 52 years, which probably corresponds to a high of patient's population already menopaused.

Our study shows that the housewives had a high risk of longer patient delay. This may be explained by the fact that housewives don't have independent resources and decision making for consulting depends largely on their husbands. This is even truer considering that the majority of our population has a lower socioeconomic level, low social security coverage and is illiterate.

Women who had gynecological “bleeding” as the first symptom were at lower risk for long “Patient delay” than the women with other first symptoms. This can be explained by the fact that the gynecological bleeding is perceived usually more urgent and more serious than gynecological infection or pelvic pain. Thus, it represents a pattern of consultation faster than other gynecological signs.

The frequency of long "Medical delay" was greater in rural than urban areas. This can be explained by the existence of a great inequality in the distribution of health care between rural and urban areas in Morocco. Morocco has one physician for 8,296 inhabitants in urban vs. one for 11,835 in rural areas [17] and the average number of consultation per capita in one year is 0.7 in urban vs. 0.4 in rural areas [18]. This inequality relates to the distribution of general practitioners and specialists, but also in particular of biological and radiological facilities [19]. This can be further explained by the difference in socioeconomic status between urban and rural areas in Morocco. Indeed, the relative poverty rate in 2007 was 14.5% in rural areas vs. 4.8% in urban areas [20].

The frequency of long total delay rate was higher among illiterate patients. This can be explained by the fact that women who have been sent to school are more conscious and can be better informed about the importance of consulting at the onset of gynecologic symptoms. They are also more aware of the importance of having additional tests as prescribed by their doctor.

The frequency of high "Total delay" was greater in rural than in urban areas. As for medical delay this may be also explained by inequality in the distribution of health care provision in Morocco and by the difference in socioeconomic status between urban and rural areas in Morocco [17–20].

The main limitation of our study is the lack of information to evaluate the doctors or patients conduct. Indeed, the "Medical delay" is composed of two parts; first part depends on the doctor′s competence and ability to follow the correct course of action and the second depends on the conduct of the patient to carefully follow the doctor′s recommendations particularly with regard to diagnostic tests.

## Conclusion

This study showed mainly a problem of late diagnosis of cervical cancer and allowed to identify factors associated with different indicators of delay before diagnosis of cervical cancer. Despite the limitation of our study, the results may represent an important tool in guiding the actions and measures of early diagnosis of cervical cancer in Morocco.
